# Functional and anatomical connectivity‐based parcellation of human cingulate cortex

**DOI:** 10.1002/brb3.1070

**Published:** 2018-07-24

**Authors:** Fei Jin, Pengpeng Zheng, Huaigui Liu, Hui Guo, Zhihua Sun

**Affiliations:** ^1^ Department of Radiology Tianjin Medical University General Hospital Tianjin China

**Keywords:** anatomical connectivity, cingulate cortex, functional connectivity, magnetic resonance imaging, parcellation

## Abstract

**Introduction:**

Human cingulate cortex (CC) has been implicated in many functions, which is highly suggestive of the existence of functional subregions.

**Methods:**

In this study, we used resting‐state functional magnetic resonance imaging (rs‐fMRI) and diffusion tensor imaging (DTI) to parcellate the human cingulate cortex (CC) based on resting‐state functional connectivity (rsFC) patterns and anatomical connectivity (AC) patterns, to analyze the rsFC patterns and the AC patterns of different subregions, and to recognize whether the parcellation results obtained by the two different methods were consistent.

**Results:**

The CC was divided into six functional subregions, including the anterior cingulate cortex, dorsal anterior midcingulate cortex, ventral anterior midcingulate cortex, posterior midcingulate cortex, dorsal posterior cingulate cortex, and ventral posterior cingulate cortex. The CC was also divided into ten anatomical subregions, termed Subregion 1 (S1) to Subregion 10 (S10). Each subregion showed specific connectivity patterns, although the functional subregions and the anatomical subregions were internally consistent.

**Conclusions:**

Using different model MRI images, we established a parcellation scheme, which is internally consistent for the human CC, which may provide an in vivo guide for subregion‐level studies and improve our understanding of this brain area at subregional levels.

## 1 INTRODUCTION

The cingulate cortex (CC) is a complex human central structure that includes the Brodmann areas 23, 24, 25, 29, 30, 31, 32, and 33 (Brodmann, 1909). The CC is implicated in a diverse array of functions, such as emotion (George et al., 1995; Kross, Davidson, Weber, & Ochsner, 2009; Liotti et al., 2000; Rogers et al., 2004; Rolls, Grabenhorst, & Parris, 2008; Walter et al., 2008, 2009), movement (Beckmann, Johansen‐Berg, & Rushworth, 2009; Paus, 2001; Picard & Strick, 1996, 2001), self‐relevant assessment (Kelley et al., 2002; Vogt, Vogt, & Laureys, 2006), cognition (Luo et al., 2007; Pourtois et al., 2010; Sohn, Albert, Jung, Carter, & Anderson, 2007; Ursu, Clark, Aizenstein, Stenger, & Carter, 2009), memory (Maguire, 2001), visuospatial orientation (Vogt et al., 2006), navigation, imagination, and planning for the future (Vann, Aggleton, & Maguire, 2009).

These diverse functions highly indicate the existence of subdivisions in CC. Regional specialization within the CC has been studied for over a century. Brodmann (1909) first defined the anterior and posterior cingulate cortex. However, these two subregions are heterogeneous in terms of their cytoarchitecture, neural pathways, and task‐related activations. Subsequently, Vogt established a widely accepted four‐region model on the basis of integrated neurobiological version (Vogt, 2005).

Yu et al. (2011) manually drew CC subregions according to the cytoarchitecture and verified that each subregion had different resting‐state functional connectivity (rsFC) patterns. However, this approach does not parcellate the CC subregions directly from the perspective of the whole‐brain connection. Alternatively, parcellation based on connectivity with other areas can provide comprehensive information to deepen our understanding of the structural and functional specializations of a specific brain region. Beckmann et al. (2009) divided the CC into nine subregions applying diffusion tensor imaging (DTI) and clarified the functional specialization of the subregions indirectly by means of meta‐analysis. Torta, Costa, Duca, Fox, & Cauda (2013) parcellated the cingulate cortex into three clusters using the results of a meta‐analytic study involved in active tasks and of three experimental studies. Neubert, Mars, Sallet, & Rushworth (2015) used functional magnetic resonance imaging (fMRI) in monkeys and humans to delineate the functional interactions of “decision‐making regions” including anterior cingulate cortex with other areas in the brain. Balsters, Mantini, Apps, Eickhoff, & Wenderoth (2016) used a combination of structural information, task‐independent, and task‐dependent functional connectivity (meta‐analytic connectivity modeling) to partition the cingulate cortex into six subregions in autism spectrum disorder (ASD). Fan et al. (2016) designed a connectivity‐based parcellation framework that identifies the subdivisions of the entire human brain which named CC subregions but the description was a few. Glasser et al. (2016) produced a population‐based 180‐area per hemisphere human cortical parcellation using multimodal data from hundreds of subjects aligned using an improved areal feature‐based cross‐subject alignment method. Consequently, regional heterogenicity in CC has been found in wealth of studies based on anatomy, function, and connectivity in human and primate. However, the CC parcellation results may not correspond completely between different studies, and it is difficult to ascertain whether these subregions constitute anatomical and functional unities. This may lead to a confounded interpretation of results related to CC subregion‐level and inconsistent findings across a number of studies and in a variety of clinical populations.

To determine functional subregions and anatomical subregions directly from the perspective of whole‐brain connection, this study used resting‐state functional magnetic resonance imaging (rs‐fMRI) and DTI from the same subjects to parcellate the human CC based on rsFC patterns and anatomical connectivity (AC) patterns. Then, we further validated whether the parcellation results obtained by the two methods were consistent.

## 2 MATERIALS AND METHODS

### 2.1 Subjects

Forty‐seven volunteers (29 males, age span 20–40 years) were included in our experiment. No participant present history of neurological or psychiatric disorders. This experiment was approved by the Ethics Committee at Tianjin Medical University General Hospital. Each participant signed a written informed consent form after given complete description of the study.

### 2.2 MRI data acquisition

All MRI measurements were performed on a 3.0‐Tesla MR scanner (Discovery MR750, GE, Milwaukee, WI, USA). Sagittal 3D T1‐weighted data were obtained by a brain volume (BRAVO) sequence, and the parameters were as follows: repetition time (TR) = 8.2 ms; echo time (TE) = 3.2 ms; field of view (FOV) = 256 × 256 mm^2^; matrix = 256 × 256; number of slices = 188; slice thickness = 1.0 mm, no gap. The rs‐fMRI images were acquired by a gradient‐echo single‐shot echo planar imaging sequence (GRE‐SS‐EPI), and the parameters: TR = 2,000 ms, TE = 45 ms; slice thickness = 4.0 mm, gap = 0.5 mm; FOV = 220 × 220 mm^2^; matrix = 64 × 64; 32 axial slices; 180 volumes. The DTI data were obtained using a GRE‐SS‐EPI sequence, and the parameters were as follows: TR = 15,000 ms; TE = 73 ms; FOV = 256 × 256 mm^2^; matrix = 128 × 128; slice thickness = 2.0 mm, no gap; number of slices = 69 axial; 50 noncollinear diffusion gradients (b = 1,000 s/mm^2^) and three nondiffusion‐weighted images (b = 0 s/mm^2^). During magnetic resonance imaging scans, participants were instructed to stay as motionless as possible and keep relaxed but not to fall asleep.

### 2.3 Definition of regions of interest

The CC mask was delineated manually in the Montreal Neurological Institute (MNI) space and wrapped back to the individual native space. The mask was checked on the sagittal planes slice by slice considering the variability of cingulate and paracingulate sulcus on morphometry and to ensure the minimum invasion to the white matter. The boundaries of CC were defined according to the descriptions provided in a previous study (Beckmann et al., 2009). The ventral boundaries of the region of interest (ROI) were the corpus callosum and rostral sulcus (Paus, Otaky, et al., 1996; Paus, Tomaiuolo, et al., 1996). The paracingulate sulcus (when present) was considered as the anterior and dorsal limit of the ROI. When the paracingulate sulcus was absent, the dorsal bank of the cingulate sulcus was considered as the dorsal boundary of the ROI at anterior positions and at more posterior levels (Paus, Otaky, et al., 1996; Paus, Tomaiuolo, et al., 1996). The dorsal boundary of the ROI followed a line that was imagined between neighboring portions of the cingulate sulcus, whenever the cingulate sulcus appeared break. The dorsal boundary of the ROI posterior to the marginal sulcus was the subparietal sulcus. In this area, the dorsal border of the ROI was a line that drawn by imagination between the anterior boundary of the subparietal sulcus and the posterior boundary of the cingulate sulcus when the two sulci were not converged. The posterior border of the ROI was a line that drawn by imagination along the shortest way between the nearest dot on the corpus callosum and the posterior limit of the subparietal sulcus. On the sagittal planes, the cingulate or paracingulate sulcus disappears laterally, and we chose the sulcus as the lateral boundary to include the entire cingulate and, the paracingulate sulci (if applicable). The mask can extend to *x* = ±10 in some subjects.

### 2.4 Parcellation of the human CC based on rsFC

#### 2.4.1 rs‐fMRI data preprocessing

The rs‐fMRI images were preprocessed using DPARSF (Data Processing Assistant for Resting‐State fMRI) and SPM8 (Statistical Parametric Mapping, http://www.fil.ion.ucl.ac.uk/spm) software package. We discarded the first 10 volumes from each subject to allow for magnetization equilibrium. Then, the remaining 170 volumes of each individual subject were corrected for differences in acquisition time between slices. After that, estimation of the head motion parameters was done. Data of the subject was omitted if the maximum rotation exceeded 2.0° or the maximum displacement exceeded 2 mm. No subjects were excluded on the basis of the criteria. Smoothing was performed on each image using a Gaussian kernel of 6 × 6 × 6 mm^3^ with full width at half maximum. Then, several parameters of spurious variance were regressed out including the linear drift, the estimated motion parameters, and the average BOLD signals in the ventricular and white matter regions. Finally, all the data were performed temporal bandpass filtering (0.01–0.08 Hz).

The skull‐stripped T1‐weighted structural images were coregistered to each subject's mean functional image, which resulted in a group of coregistered T1 images in fMRI space. Then, the T1 images in fMRI space were normalized to the MNI space. Next, we transformed the CC ROI from MNI space into each individual fMRI space using the inverted transformation parameters. Finally, the ROI of every subject in native rs‐fMRI space was acquired.

#### 2.4.2 ROI‐based cross‐correlation

The whole‐brain rsFC for each CC ROI voxel of each subject was computed using Pearson correlation coefficients and then converted to z values using Fisher's r‐to‐z transformation. Cross‐correlation was calculated between the rsFC patterns of all voxels in ROI and applied for automatic parcellation (Johansen‐Berg et al., 2004).

#### 2.4.3 FC‐based parcellation

We fed the correlation matrix into the K‐means clustering algorithm and grouped voxels that share similar connection profiles in the seed region with other voxels of the brain. In this study, the experimenter determined the number of K‐means cluster first. We used cross‐validation to determine the number of clusters that yielded optimal consistency across subjects and, hence, the optimal number of clusters. Specifically, we used a leave‐one‐out method in which each subject's data were excluded from the averaging.

For each subject, we checked the consistency between the clustering results of the single subject and the average across the remaining subjects using Cramer's *V*. Cramer's *V* is a measure of association between two nominal variables and it is calculated based on chi‐square (*χ* ^2^) statistic(Cramer, 1946). As an example of 2 clusters, one variable is the category of clusters (*r* = 2) of a single subject and another variable is the category of the average clusters (*c* = 2) across the remaining subjects. According to the frequency distribution of the two variables for each voxel of interest, a 2 × 2 contingency table was obtained. *V* is calculated by first calculating chi‐square, then using the following calculation: [*V* = SQRT(*χ* ^2^/(*n*/(*k* − 1)))]. Where: chi‐square is derived from Pearson's chi‐square test; *n* is the grand total number of voxels of interest; and k is the number of rows or the number of columns, whichever is less. Cramer's *V* gives values within the interval (0, 1) where high values indicate good consistency with a value of 1 indicating a perfect match(Li et al., 2013). The intersubject consistency was checked for *k* = 3–12 clusters.

The maximum probability map (MPM) was calculated to exhibit the final results (Caspers et al., 2008). In this process, all 47 parcellation results from individual space were transformed to the MNI space. We calculated the MPM by assigning each voxel to the cluster in which it was most likely to be located.

#### 2.4.4 Whole‐brain rsFC pattern of each CC functional subregion

We extracted the mean time series of the CC subregions from the four‐dimensional residual time series data. To improve the normality, Pearson correlation coefficients between the mean time series of each subregion and that of each voxel of the whole brain were computed and converted to *z* values using Fisher's *r*‐to‐*z* transformation for each subject. Next, a random‐effect one‐sample *t* test was applied in a voxelwise manner to identify brain regions that showed correlations significantly with the subregion. All results were corrected for familywise error (FWE) of multiple comparisons with a threshold of *p* < 0.05 and a cluster size of >50 voxels.

### 2.5 Parcellation of the human CC based on AC

#### 2.5.1 DTI data preprocessing

Two radiologists were in charge of visually inspecting the DTI and T1‐weighted data for abnormal brain features and scanner artifacts. Simple head motions and eddy‐current distortion were corrected with FSL 4.0 (FMRIB Software Library, http://www.fmrib.ox.ac.uk/fsl).

The skull‐stripped T1‐weighted structural images were coregistered to each subject's B0 images using SPM8, which generated a group of coregistered T1 images in DTI template. Then, the resulting images were normalized to the MNI space. Subsequently, we transformed the CC ROI from MNI space into each individual diffusion space using the inverted transformation parameters. Finally, the ROI of each subject in native diffusion space was acquired.

#### 2.5.2 Probabilistic tractography

Probabilistic fiber tracking was performed using the FSL package. Estimations in voxelwise of the fiber probability distributions were generated by Bedpostx. For each voxel, probability distributions were calculated in two fiber directions using multiple fiber extension (Behrens, Berg, Jbabdi, Rushworth, & Woolrich, 2007) according with a diffusion modeling approach previously published (Behrens, Johansen‐Berg, et al., 2003; Behrens, Woolrich, et al., 2003). We calculated connection probability between each voxel in CC ROI and the other voxels in whole brain by computing the number of traces reaching into the target site. We thresholded the Probtrack estimates using a connection probability of *p* < 0.002 (10 of 5,000 samples) to reduce false‐positive connections.

#### 2.5.3 AC‐based parcellation

Similar to the above FC‐based parcellation, the correlation matrix was fed into a K‐means clustering algorithm for automatic clustering. We also performed cross‐validation to determine optimal number of clusters and calculated the MPM to show the final results.

#### 2.5.4 Whole‐brain AC pattern of each CC anatomical subregion

To elucidate the whole‐brain AC pattern for each CC subregion, we transformed the anatomical subregions to diffusion space, and then Probtracking (Behrens et al., 2007) for each voxel in each subregion was run by estimating fiber orientations. The identified fiber tracts were transformed into MNI space, and then we computed an average map for each CC subregion.

We also performed fingerprints to analyze the probabilistic connections between each CC subregion and target regions. The target regions were defined by the automated anatomical labeling (AAL) atlas (Tzourio‐Mazoyer et al., 2002) that divided each cerebral hemisphere into 45 brain regions. Forty‐two brain regions on each cerebral hemisphere except for the bilateral anterior cingulate cortex (ACC), midcingulate cortex (MCC), and posterior cingulate cortex (PCC) were defined as target regions. Diffusion tensor tractography (DTT) was used to estimate the averaged connection probability between each subregion of the CC and every region of the 42 target regions within the same hemisphere. Finally, we got the top five brain regions that showed the highest connection probability with each CC subregion. After removing the repetitive regions, we finally obtained 14 target regions in the left CC and 13 target regions in the right CC. The difference between the bilateral CC target regions was that the calcarine and superior occipital gyrus was only identified as a target region of the left CC while frontal superior cortex was only identified as a target region of the right CC. Considering the value of connection probability to frontal superior cortex in right CC was very weak, and to compare the fingerprints of the bilateral CC, we computed the fingerprint of each CC subregion with the same 14 left target regions.

#### 2.5.5 Comparison between functional subregions and anatomical subregions

From the perspective of anatomical location and connectivity patterns, we explored whether there was consistency between functional subregions and anatomical subregions. At the same time, we calculated the matrices of parcel correlation coefficients (*r*) between the parcellation results based on rs‐fMRI and DTI. The parcel *r*‐matrices were used to study parcel‐to‐parcel correlations, and high values indicate good consistency.

## 3 RESULTS

### 3.1 Functional subregions and anatomical subregions of CC

**Figure 1:**
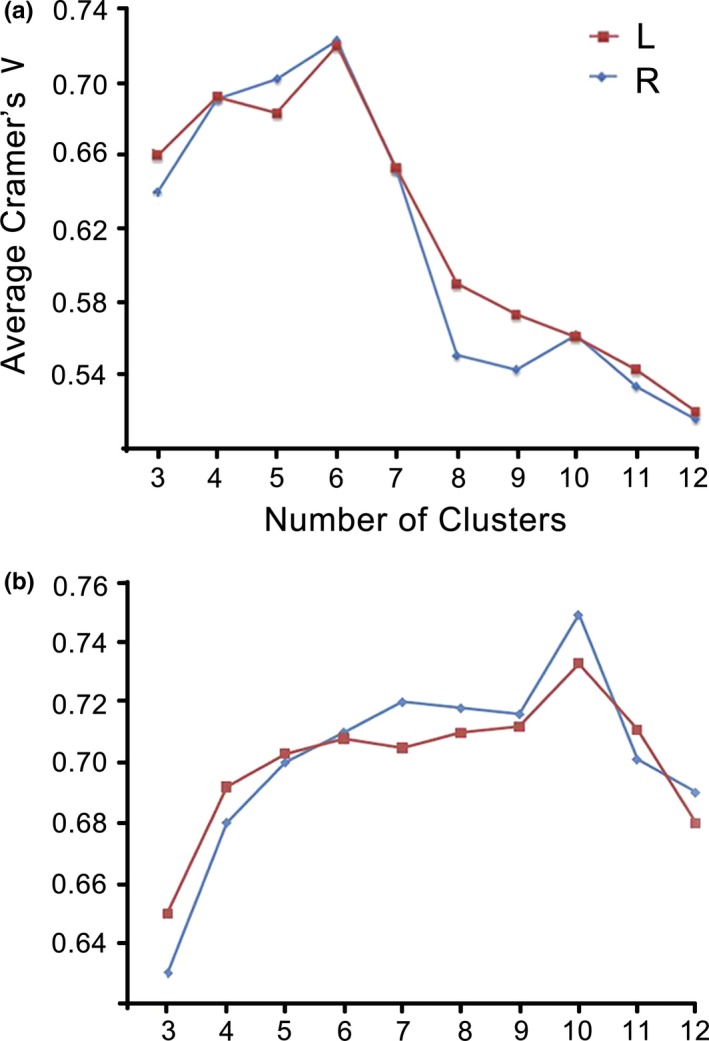
Average Cramer's *V* as an indication of clustering consistency in CC (a: FC‐based parcellation; b: AC‐based parcellation)

The present study utilized rsFC patterns and AC patterns to parcellate the human CC and determined whether a corresponding topography exists. We selected the number of clusters *k* = 3–12 to calculate the Cramer's *V* values. Finally, we found that the optimal number of clusters for the CC was estimated to be 6 in FC‐based parcellation, which provided the highest consistency of clustering across subjects, while ten clusters provided the highest consistency in anatomical parcellation (Figure 1).

**Figure 2:**
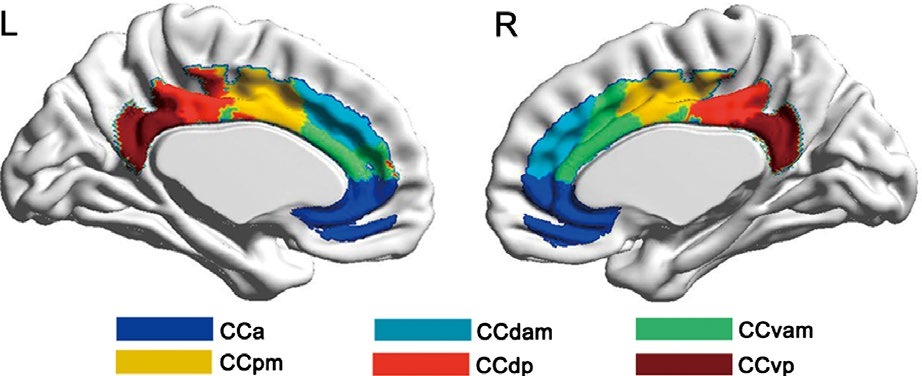
FC‐based parcellation of the human CC. CCa, anterior cingulate cortex; CCdam, dorsal anterior midcingulate cortex; CCdp, dorsal posterior cingulate cortex; CCpm, posterior midcingulate cortex; CCvam, ventral anterior midcingulate cortex; CCvp, ventral posterior cingulate cortex; FC, functional connectivity

**Figure 3:**
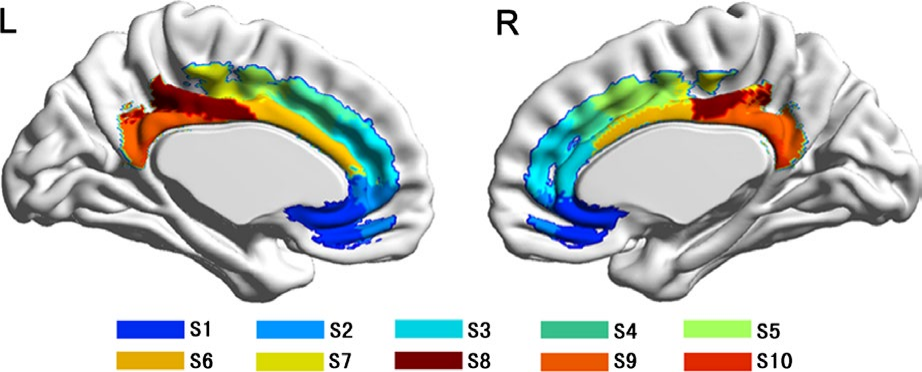
AC‐based parcellation of the human CC. AC, anatomical connectivity; S, subregion

From the MPM, we can identify six functional subregions of the CC: the anterior cingulate cortex (CCa), dorsal anterior midcingulate cortex (CCdam), ventral anterior midcingulate cortex (CCvam), posterior midcingulate cortex (CCpm), dorsal posterior cingulate cortex (CCdp), and ventral posterior cingulate cortex (CCvp) (Figure 2, Figure S1). Based on AC patterns, we parcellated the CC into ten anatomical subregions, termed subregion 1 to subregion 10 (Figure 3, Figure S2).

### 3.2 Whole‐brain rsFC patterns of CC functional subregions

**Figure 4:**
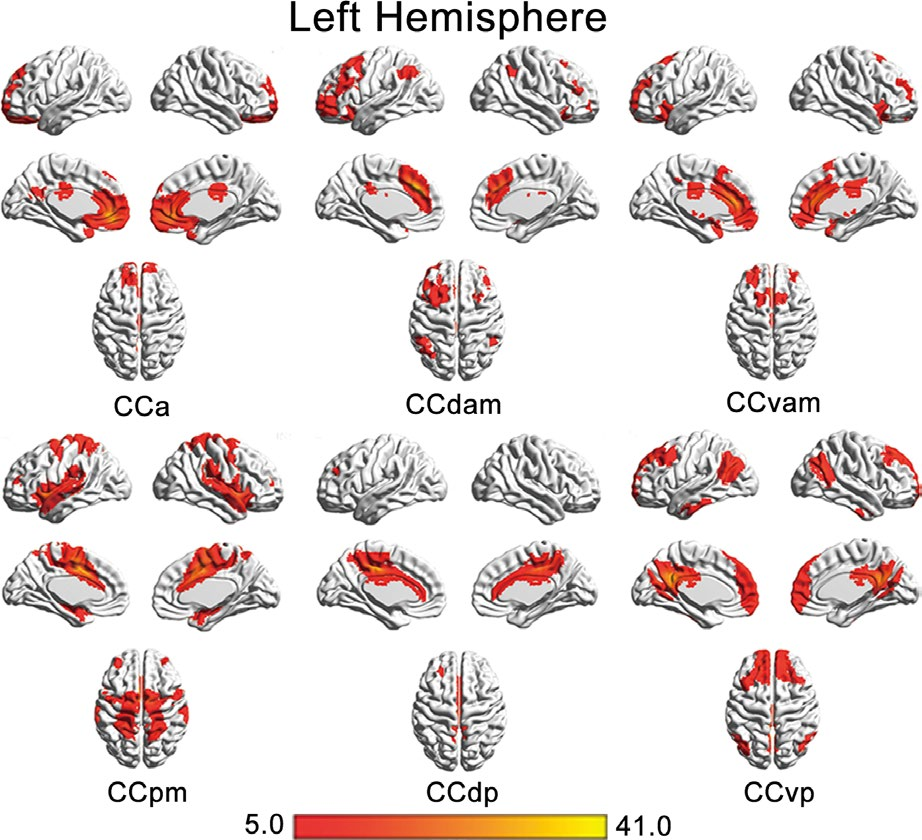
Whole‐brain functional connection patterns of the left CCa, CCdam, CCvam, CCpm, CCdp, CCvp subregions. CCa, anterior cingulate cortex; CCdam, dorsal anterior midcingulate cortex; CCdp, dorsal posterior cingulate cortex; CCpm, posterior midcingulate cortex; CCvam, ventral anterior midcingulate cortex; CCvp, ventral posterior cingulate cortex

The whole‐brain rsFC for each CC subregion was calculated to identify its involved cortical network. The brain regions which each CC functional subregion was connected to were delineated using AAL atlas. The whole‐brain rsFC map of each CC subregion was displayed on a three‐dimensional brain surface template using the BrainNet software (Figure 4, Figure S3). Considering debate remains in the significance of the negative rsFC patterns, we focused only on the positive rsFC patterns of each subregion.

The CCa was related to the superior frontal gyrus (SFG), medial prefrontal cortex (MPFC), orbital frontal cortex (OFC), insular cortex, precuneus, temporal pole, MCC, PCC. The CCdam was related to the supplementary motor area (SMA), premotor area, dorsolateral prefrontal cortex (DLPFC), SFG, middle frontal gyrus (MFG), MPFC, OFC, angular gyrus (AG), and PCC. The CCvam was related to the MPFC, frontal‐insular cortex, DLPFC, premotor area, SMA, supramarginal gyrus (SMG), DLPFC, premotor area, SMA, Rolandic area, temporal lobe, and ACC. The CCpm was related to the precentral gyrus, postcentral gyrus, premotor area, paracentral lobule, SMA, SMG, Rolandic area, insula lobe, inferior frontal gyrus (IFG), temporal pole, superior temporal gyrus (STG), middle temporal gyrus (MTG), and precuneus. The CCdp was related to the SFG, MFG, IFG, precuneus, AG, MCC, SMG, and paracentral lobule. The CCvp was related to the SFG, MFG, MPFC, precuneus, AG, MTG, and inferior temporal gyrus (ITG).

### 3.3 Whole‐brain AC patterns of CC anatomical subregions

**Figure 5:**
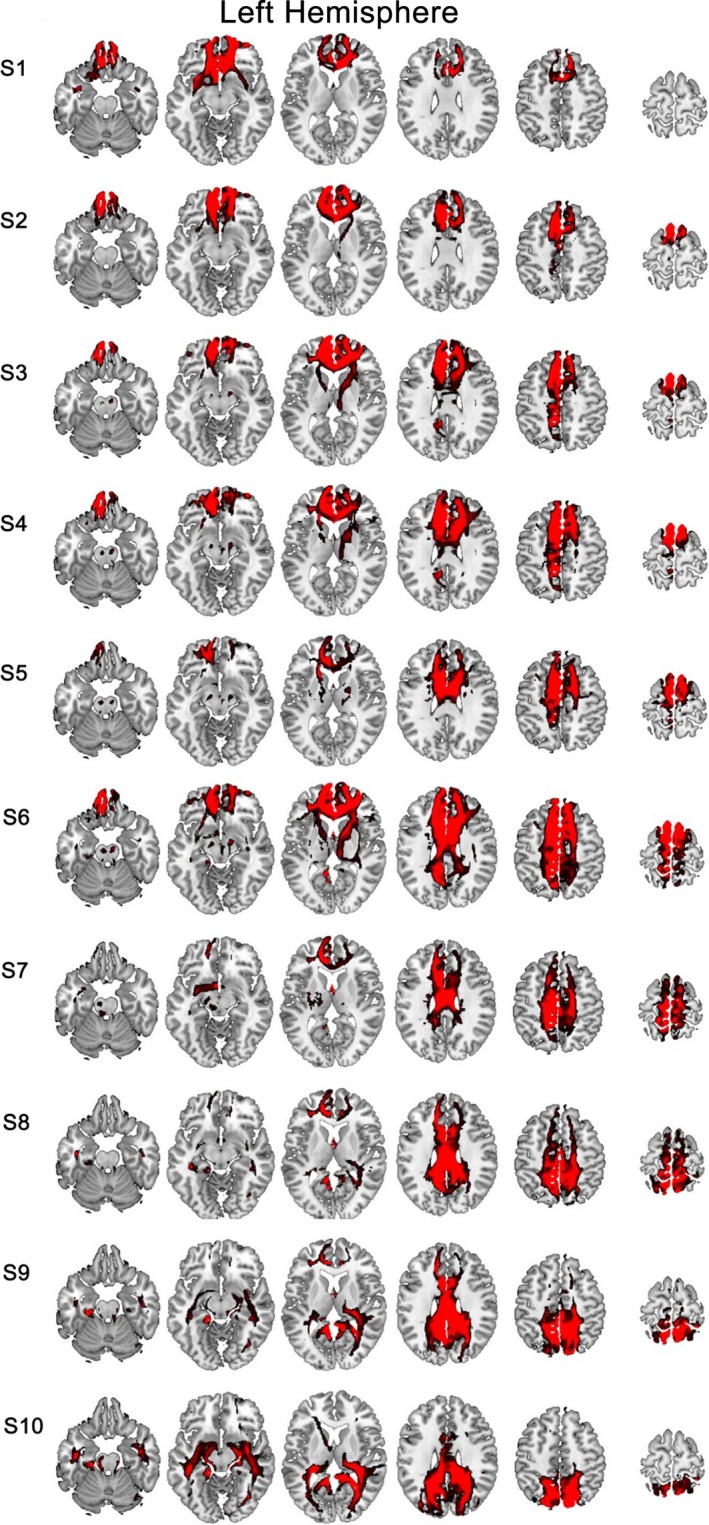
Whole‐brain anatomical connection patterns of the left S1–S10

The brain regions which each CC anatomical subregion was connected to were delineated using the AAL atlas. The primary anatomical connections for subregion 1 were in the SFG, MPFC, OFC, insular lobe, temporal pole, MCC, and corpus callosum. The AC patterns of subregion 2 were primary to the SMA, SFC, MPFC, OFC, MCC, and corpus callosum. Subregions 3 and 4 were both primarily connected to the SMA, DLPFC, SFC, MFC, MPFC, OFC, anterior limb of the internal capsule, thalamus, ACC, PCC, and corpus callosum. Subregion 5 was connected to the paracentral lobule, SMA, anterior limb of internal capsule, thalamus, ACC, corpus callosum, MPFC, and DLPFC. The primary anatomical connections for subregion 6 were the paracentral lobule, SMA, anterior limb of internal capsule, thalamus, MPFC, OFC, SFC, MFC, precuneus, MCC, and corpus callosum. Subregion 7 was connected to the SFC, MFC, paracentral lobule, precuneus, SMA, anterior limb of internal capsule, thalamus, ACC, and MCC. Subregion 8 was connected to the SFC, MFC, paracentral lobule, precuneus, ACC, and MCC. The AC patterns of subregion 9 were primary to the SFC, MFC, precuneus, temporal lobe, ACC, MCC, and corpus callosum. Subregion 10 was connected to the precuneus, temporal lobe, MCC, and corpus callosum (Figure 5, Figure S4).

**Figure 6:**
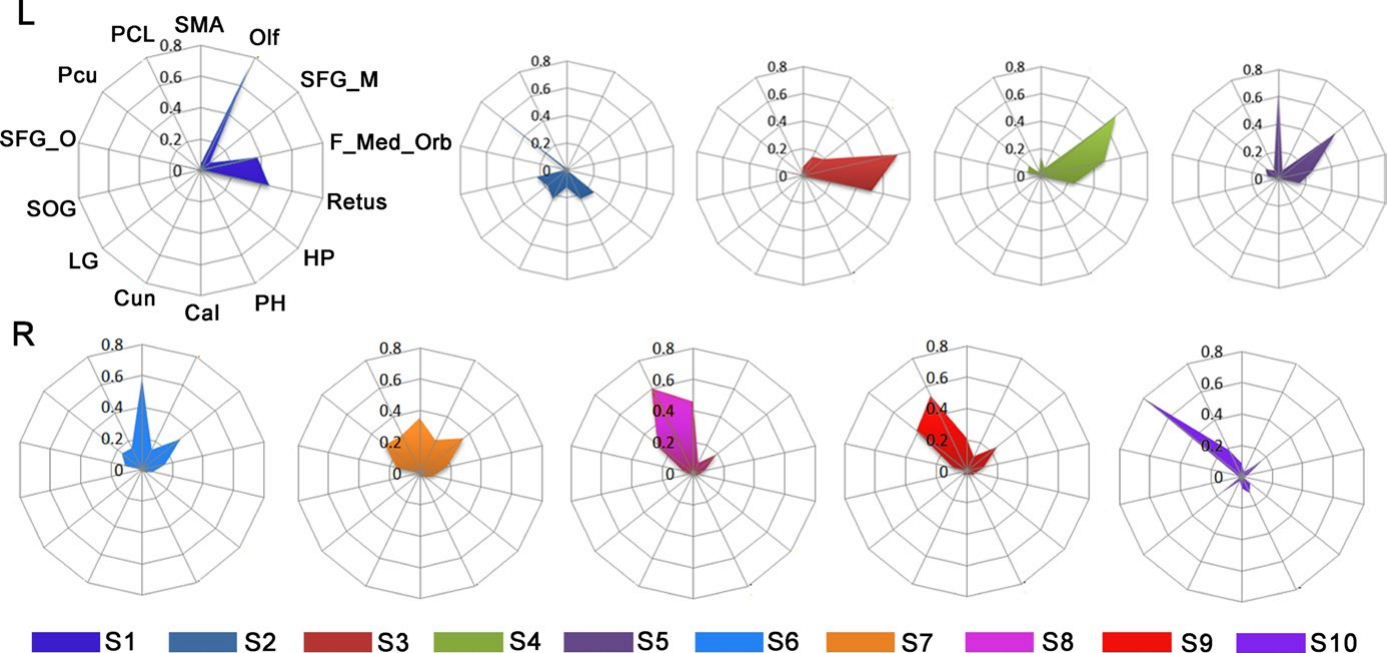
The fingerprints (L: left; R: right) of the CC subregions. They are shown in different colors. Cal, calcarine; Cau, caudate; F_Med_Orb, frontal medial orbital cortex; HP, hippocampus; LG, lingual gyrus; Olf, olfactory; PCL, paracentral lobule; Pcu, precuneus; PH, parahippocampal; Rectus, rectus; SFG_M, middle superior frontal gyrus; SFG_O, orbital part of the superior frontal gyrus; SMA, supplementary motor area; SOG, superior occipital gyrus

The fingerprint method was used to directly compare the anatomical connectivity patterns of each CC subregion (Figure 6, Figure S5). The main anatomical connections for subregion 1 were olfactory, rectus and frontal medial orbital cortex. The connection strength between subregion 2 and seed regions was weak. The AC areas in subregion 3 and subregion 4 were similar; however, subregion 3 was mainly connected to the frontal medial orbital cortex, while subregion 4 was more connected to the middle superior frontal gyrus. Both subregion 5 and 6 were mainly connected to SMA and middle superior frontal gyrus. The connection strength between subregion 7 and seed regions were weak. Both subregion 8 and 9 were mainly connected to paracentral lobule. Subregion 10 was mainly connected to precuneus.

### 3.4 Comparison between functional subregions and anatomical subregions

We explored the correlation between functional subregions and anatomical subregions from the perspective of anatomical location and connectivity patterns.

Subregion 1 (areas 25, 24, s32, 33) and subregion 2 (areas p32, d32, 24c) represented the CCa. Subregions 3 and 4 represented the CCdam (areas 32′, a24c’). Subregion 5 (areas p24c’, 24d) and the posterior part of subregion 6 (areas p24′, p33′) represented the CCpm. The anterior part of subregion 6 (areas a24′, a33′) represented the CCvam. Subregion 7 (area 23c) and subregion 8 (areas 23d, d23, d31) represented the CCdp. Subregion 9 (areas v23, v31) and subregion 10 (areas 29/30) represented the CCvp (Table 1).

**Table 1:** Cingulate cortex subregions based on rsFC, AC, and cytoarchitecture

rsFC	AC	Cytoarchitecture
CCa	S1	25, 24 s32, 33
	S2	p32, d32, 24c
CCdam	S3, S4	32′, a24c’
CCvam	Anterior part of S6	a24′, a33′
CCpm	S5	p24c’, 24d
	Posterior part of S6	p24′, p33′
CCdp	S7	23c
	S8	23d, d23, d31
CCvp	S9	v23, v31
	S10	29/30

The CC parcels show high spatial correlations in both left (*r* = 0.68, *p* < 0.001) and right (*r* = 0.72, *p* < 0.001) hemispheres between rs‐fMRI and DTI patterns based on the above inter‐subregion correspondence. High spatial correlations reflect the functional subregions and the anatomical subregions were internally consistent, and indicate objectively the reliability of our parcellation result.

## 4 DISCUSSION

To the best of our knowledge, this is the first study to parcellate the human CC based on functional and anatomical connection patterns respectively and to elucidate the anatomical and functional connectivity patterns of the human CC at the subregional level. The present study parcellated CC into 6 subregions in rsFC patterns and 10 subregions in AC patterns and the results demonstrated a correspondence correlation between functional subregions and anatomical subregions. We found that each cingulate subregion is specifically involved in different brain networks. These findings may improve our understanding of CC connectivity and function at the level of subregions.

### 4.1 Connectivity profiles of the CC functional subregions

The CCa was functionally correlated with the affective network (AN), including the OFC and MPFC, and the default‐mode network (DMN), including the MPFC, SFG, PCC, precuneus, and temporal pole. The subgenual part of the ACC is the repository of negative emotion where intense sadness is associated with increases in regional cerebral blood flow (Mayberg et al., 1999). In contrast, the pregenual part of the ACC is associated with positive emotion (Phan, Wager, Taylor, & Liberzon, 2002; Vogt, Berger, & Derbyshire, 2003). Although the types of emotion associated with the subgenual and pregenual ACC are different, we could not divide the ACC into positive and negative emotion areas due to the present parcellation method based on resting‐state, rather than task fMRI. The present study showed that the CCa was related to the AN. Consistent with the previous findings on the DMN (Fox et al., 2005; Fransson, 2005; Greicius, Krasnow, Reiss, & Menon, 2003), we found that the CCa showed positive FC with different brain network, which accords with the statement that the CCa is a key node of the DMN.

The MCC was divided into three functional subregions. The positive FC between each MCC subregion and sensorimotor network (SMN) may be explained by the location of cingulate motor area in the MCC (Hatanaka et al., 2003). The anterior part of the MCC (CCdam, CCvam) showed extensive FC with brain areas belonging to the SMN, including the SMA and premotor area, which is in accordance with its function in performing complex motor tasks (Picard & Strick, 1996). The CCpm showed extensive FC with the primary motor cortex, especially the precentral gyrus and paracentral lobule, which are activated during simple motor tasks (Picard & Strick, 1996). Both the CCdam and CCvam were related to the cognitive network (CN) and AN. This conclusion may be explained by a previous study that investigated dorsal ACC and MCC, showing that they subserved cognitive and emotional processing (Bush et al., 2002). Some studies found that the MCC is a core node of empathy that includes cognitive and affective components (Decety, Chen, Harenski, & Kiehl, 2013; Fan, Duncan, de Greck, & Northoff, 2011). So the contribution part may be the anterior part of the MCC (CCdam, CCvam) according to our study. Unlike the CCdam, the CCvam showed positive FC with the frontal‐insular cortex, which is a core node of the salience network.

The extensive FC between the CCpm and brain regions belonging to the perception‐ motor planning and processing system (Woods, Hernandez, Wagner, & Beilock, 2014) suggests that the CCpm is involved in perception‐cognition processing. Both the CCdp and CCvp were functionally correlated with the DMN, which is in accordance with previous rsFC findings (Tomasi et al., 2009; Yu et al., 2011). In addition to the rsFC with DMN, the CCdp was functionally correlated with the SN. This finding was consistent with a previous study showing that the PCC acts as a hub for the DMN‐SN cross‐network (Hemington, Wu, Kucyi, Inman, & Davis, 2015). Therefore, it was suggested that this “hub” is located in the CCdp.

### 4.2 Connectivity profiles of the CC anatomical subregions

Subregion 1 (areas 25, 24, s32, 33) and subregion 2 (areas p32, d32, 24c) had anatomical connections with the SFG, MPFC, OFC, insular lobe, and temporal pole, which is in accordance with previous rsFC findings (Reser et al., 2017)^.^ Subregions 3–6 (areas 32′, a24c’, p24c’, 24d, p24′, p33′) were anatomically connected to the anterior limb of the internal capsule and thalamus, which is consistent with the finding that the MCC is anatomically connected with the thalamus via the anterior thalamic radiation (Erpelding & Davis, 2013). In addition, subregions 3‐6 were also anatomically correlated with motor‐related regions, which is consistent with a previous finding showing that part of the cingulate gyrus sends projections to the primary motor cortex, premotor area and SMA (Pandya, Van Hoesen, & Mesulam, 1981). The MCC subregions also connected with the DLPFC, SFG, MFG, MPFC and OFC, which suggests that the MCC is involved in cognition and affection. Furthermore, there were differences in the connections of the bilateral MCC subregions because of lateralization (Huster, Westerhausen, Kreuder, Schweiger, & Wittling, 2007). Anatomical connections between PCC subregions and the SFG, MFG, precuneus, and temporal lobe have been reported (Ma et al., 2015).

Using magnetic resonance diffusion tractography, Beckmann et al. (2009) divided the CC into 9 subregions with distinctive connectivity profiles in 11 subjects. The position of cluster 1 plus cluster 2 by Beckman et al. is similar to the sum of subregion 1 and subregion 2 in our study. The position of the sum of cluster 8 and cluster 9 by Beckman et al. resembles subregion 9 plus subregion 10 in ours’. But the position of single subdivision from different studies is not in perfect agreement. The differences may be explained by many reasons such as subject numbers and different imaging parameters.

### 4.3 The correspondence between CC functional subregions and anatomical subregions

Although there is not a direct one‐to‐one correspondence between functional subregions and anatomical subregions, we really found certain correspondence. Subregion 1 (areas 25, 24, s32, 33) and subregion 2 (areas p32, d32, 24c) represented the CCa. Subregions 3 and 4 represented the CCdam (areas 32′, a24c’). Subregion 5 (areas p24c’, 24d) and the posterior part of subregion 6 (areas p24′, p33′) represented the CCpm. The anterior part of subregion 6 (areas a24′, a33′) represented the CCvam. Subregion 7 (area 23c) and subregion 8 (areas 23d, d23, d31) represented the CCdp. Subregion 9 (areas v23, v31) and subregion 10 (areas 29/30) represented the CCvp.

The previous study has demonstrated that anatomical connection is the neural basis of functional connectivity (Greicius, Supekar, Menon, & Dougherty, 2009). Thus, FC may reflect both direct and indirect anatomical connections between two brain regions. In other words, the different anatomical subregions may show the similar functional connectivity. That's maybe the reason why the subregion based on functional connectivity pattern corresponds to one or more subregions based on anatomical connectivity (AC) pattern.

Several limitations should be mentioned in this study. First, the imaging parameters were unconvincing, which may make our study to be underpowered. Second, there is not a gold standard for estimating the degree of homogeneity within the created parcels based on AC and FC at present. Future studies are needed to validate these interpretations of our results.

## 5 CONCLUSION

In the present study, we revealed that the human CC is a highly heterogeneous area that can be divided into six functional subregions and ten anatomical subregions. This parcellation scheme was further demonstrated by utilizing particular connectivity pattern analyses and functional characterization. These parcellation results may facilitate future clinical and subregion‐level research addressing this area.

## CONFLICT OF INTEREST

The authors declare that the research was conducted in the absence of any commercial or financial relationships that could be construed as a potential conflict of interest.

## AUTHOR CONTRIBUTIONS

FJ and PZ wrote the protocol and the draft of the manuscript text. HL and HG performed image processing and statistical analyses. FJ and HL collected the magnetic resonance imaging (MRI) data. ZS designed the experiment and revised the manuscript. All authors contributed to and have approved the final manuscript.
