# Supplementary figures and images for: Functional and anatomical connectivity‐based parcellation of human cingulate cortex
Source: Brain Behav. 2018 Jul 24;8(8):e01070. doi: 10.1002/brb3.1070 (PMC6085915; doi:10.1002/brb3.1070)

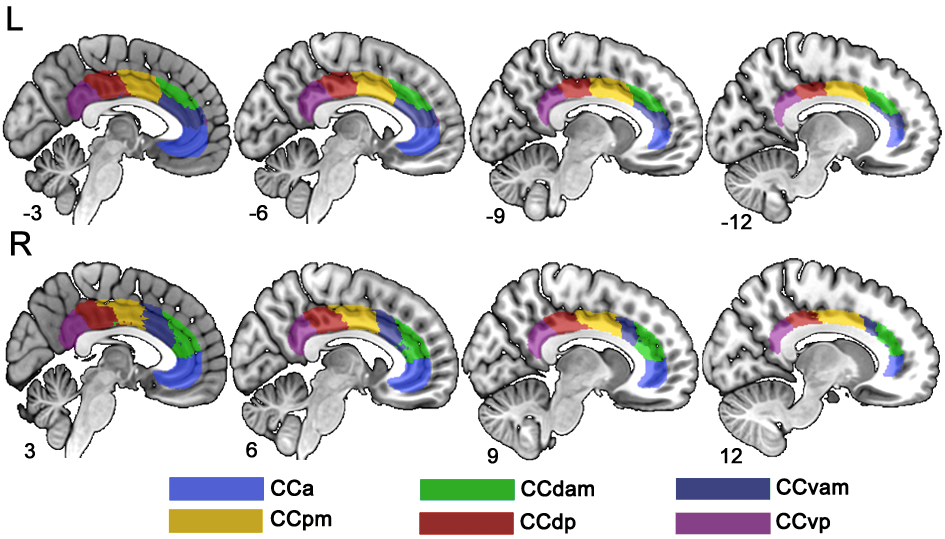

Supplement: Supplementary file 1 [file BRB3-8-e01070-s001.png]

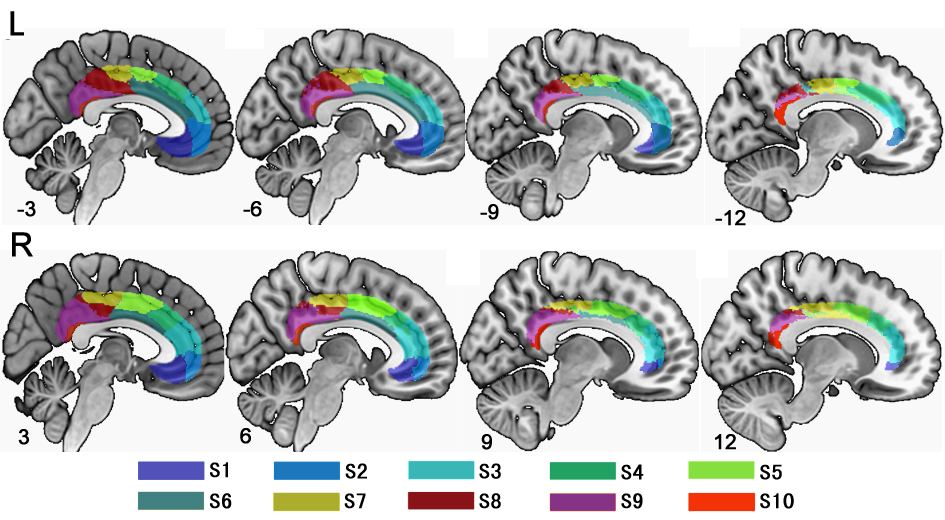

Supplement: Supplementary file 2 [file BRB3-8-e01070-s002.png]

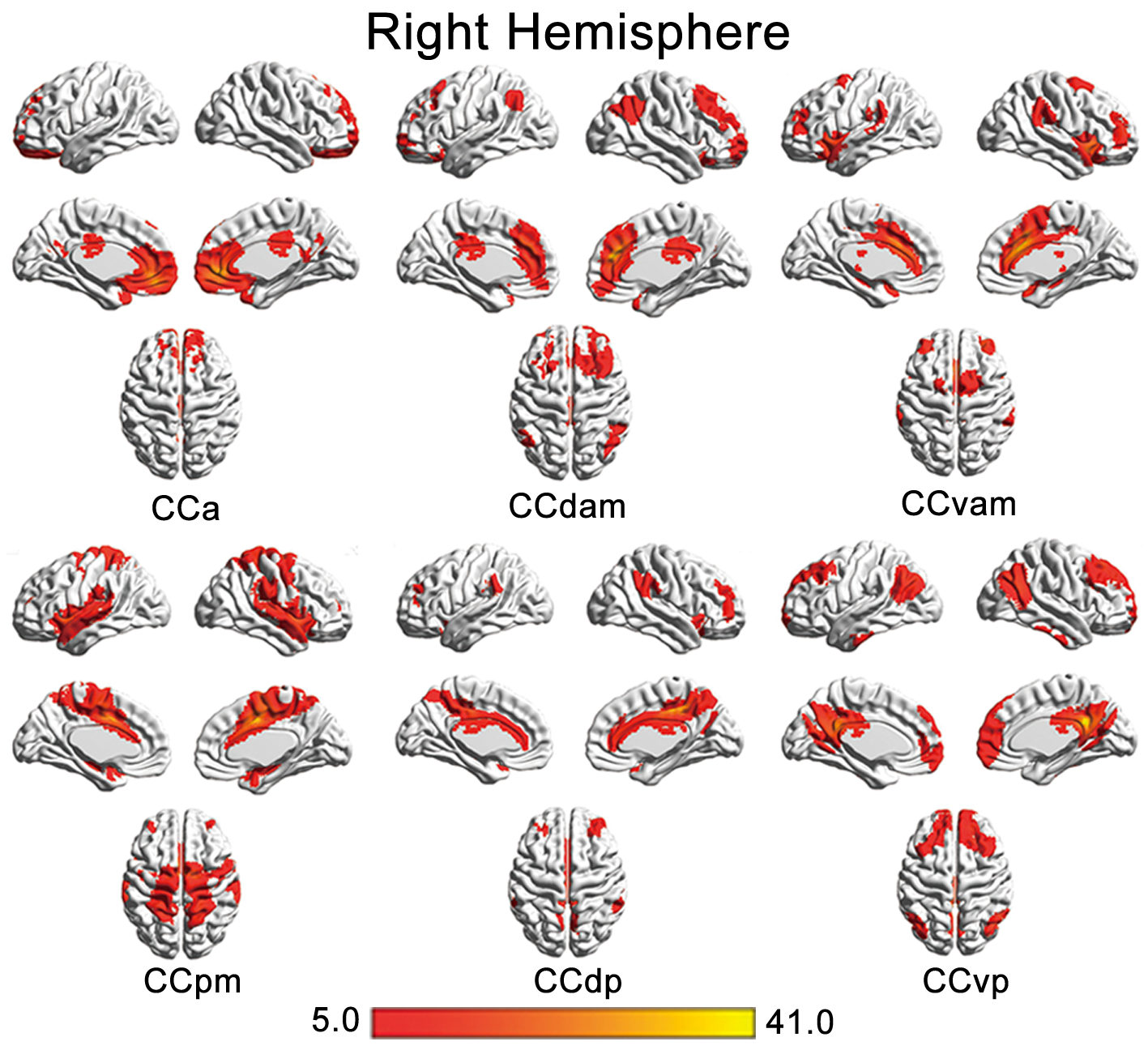

Supplement: Supplementary file 3 [file BRB3-8-e01070-s003.png]

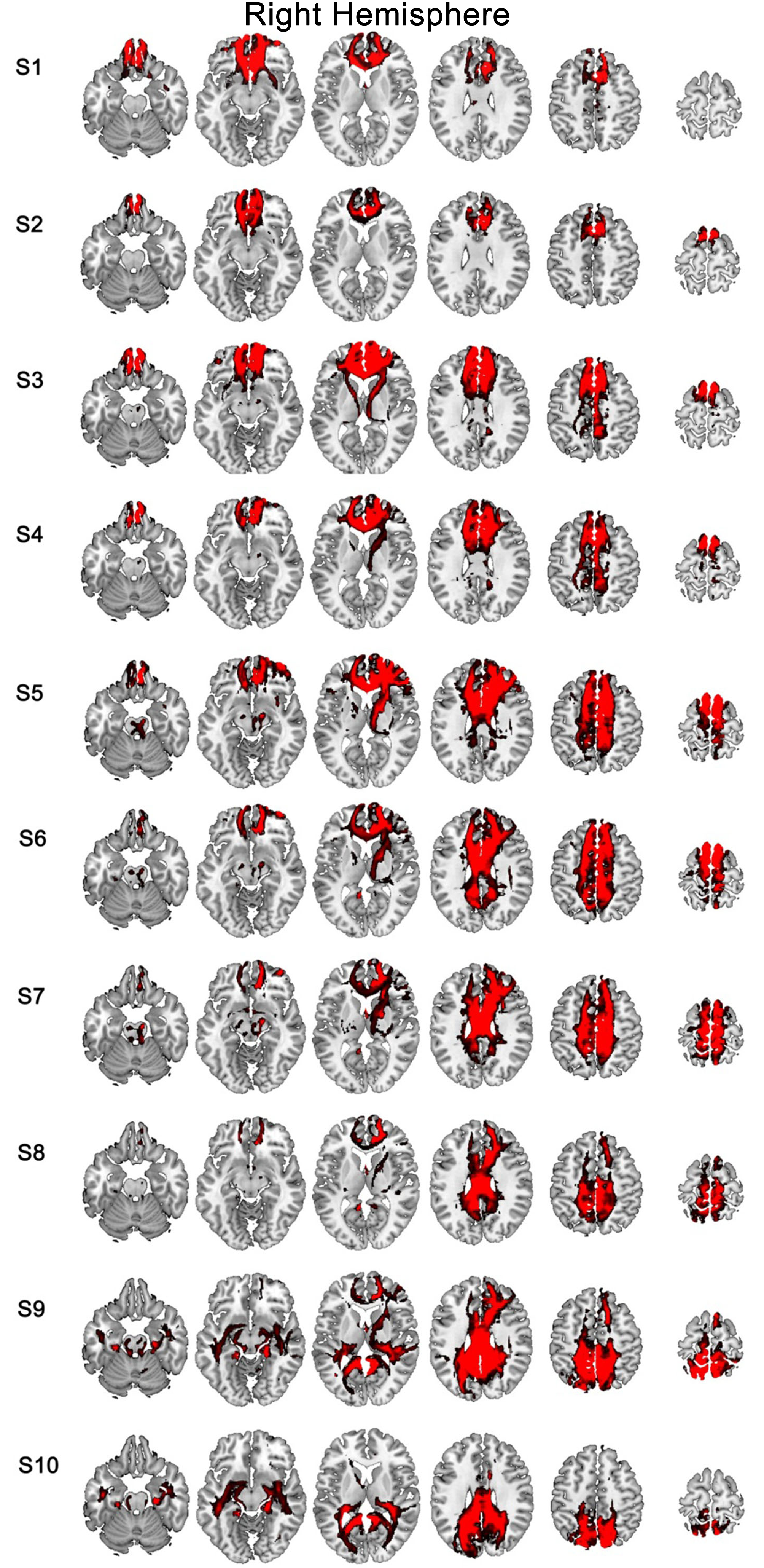

Supplement: Supplementary file 4 [file BRB3-8-e01070-s004.png]

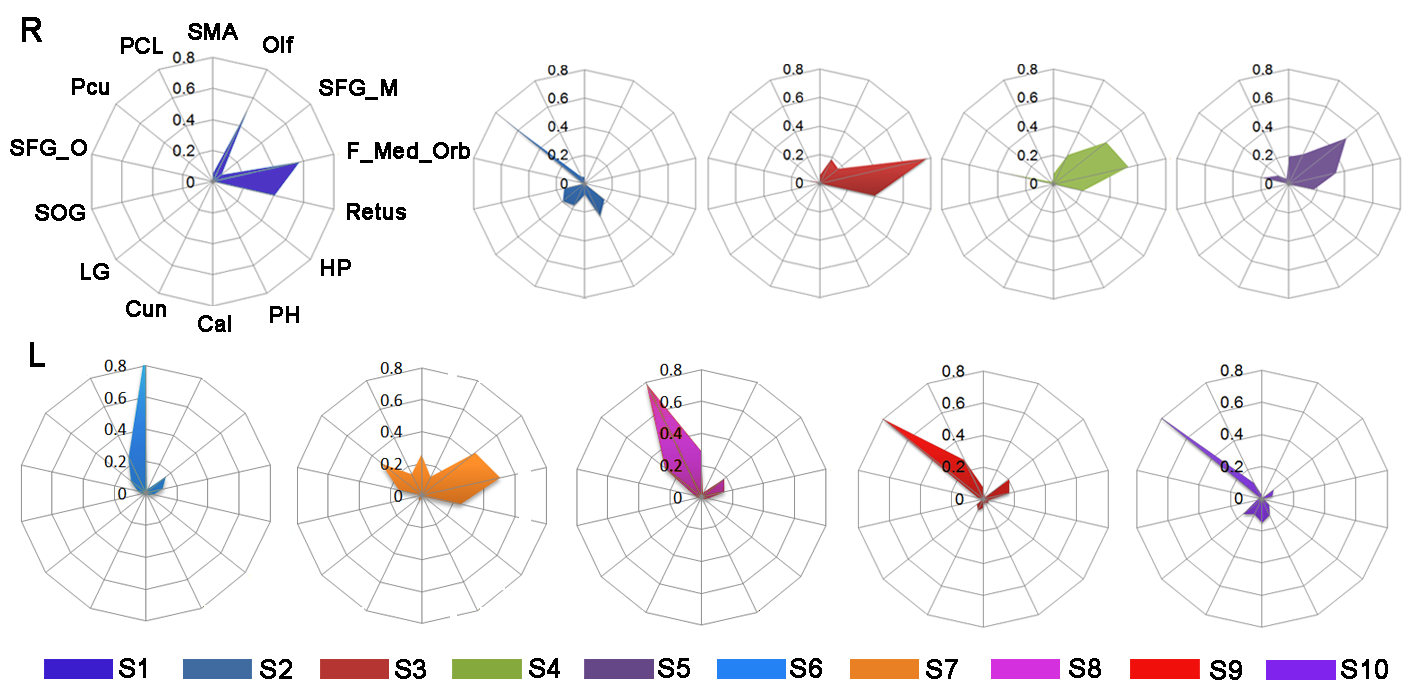

Supplement: Supplementary file 5 [file BRB3-8-e01070-s005.png]
